# Biomechanical Evaluation of Patient-Specific Polymethylmethacrylate Cranial Implants for Virtual Surgical Planning: An In-Vitro Study

**DOI:** 10.3390/ma15051970

**Published:** 2022-03-07

**Authors:** Bilal Msallem, Michaela Maintz, Florian S. Halbeisen, Simon Meyer, Guido R. Sigron, Neha Sharma, Shuaishuai Cao, Florian M. Thieringer

**Affiliations:** 1 Clinic of Oral and Cranio-Maxillofacial Surgery, University Hospital Basel, CH-4031 Basel, Switzerland; bilal.msallem@usb.ch (B.M.); neha.sharma@unibas.ch (N.S.); shuaishuai.cao@unibas.ch (S.C.); florian.thieringer@usb.ch (F.M.T.); 2 Medical Additive Manufacturing Research Group (Swiss MAM), Department of Biomedical Engineering, University of Basel, CH-4123 Allschwil, Switzerland; michaela.maintz@unibas.ch; 3 Basel Institute for Clinical Epidemiology and Biostatistics, Department of Clinical Research, University Hospital Basel, University of Basel, CH-4031 Basel, Switzerland; floriansamuel.halbeisen@usb.ch; 4 Clinic of Oral and Cranio-Maxillofacial Surgery, Cantonal Hospital Aarau, CH-5001 Aarau, Switzerland; guido.sigron@ksa.ch

**Keywords:** 3D printing, cranioplasty, fracture force, molding, porosity, template, thickness

## Abstract

Cranioplasty with freehand-molded polymethylmethacrylate implants is based on decades of experience and is still frequently used in clinical practice. However, data confirming the fracture toughness and standard biomechanical tests are rare. This study aimed to determine the amount of force that could be applied to virtually planned, template-molded, patient-specific implants (*n* = 10) with an implant thickness of 3 mm, used in the treatment of a temporoparietal skull defect (91.87 cm^2^), until the implant cracks and finally breaks. Furthermore, the influence of the weight and porosity of the implant on its force resistance was investigated. The primary outcome showed that a high force was required to break the implant (mean and standard deviation 1484.6 ± 167.7 N), and this was very strongly correlated with implant weight (Pearson’s correlation coefficient 0.97; *p* < 0.001). Secondary outcomes were force application at the implant’s first, second, and third crack. Only a moderate correlation could be found between fracture force and the volume of porosities (Pearson’s correlation coefficient 0.59; *p* = 0.073). The present study demonstrates that an implant thickness of 3 mm for a temporoparietal skull defect can withstand sufficient force to protect the brain. Greater implant weight and, thus, higher material content increases thickness, resulting in more resistance. Porosities that occur during the described workflow do not seem to reduce resistance. Therefore, precise knowledge of the fracture force of polymethylmethacrylate cranial implants provides insight into brain injury prevention and serves as a reference for the virtual design process.

## 1. Introduction

Reconstruction of the craniofacial skeleton’s highly complex shape is routinely achieved using various autogenous or alloplastic materials [1,2]. Alloplastic reconstructions using freehand-molded polymethylmethacrylate (PMMA) have been a gold standard in cranioplasty since World War II [1,3,4]. The use of PMMA in cranioplasty resulted from the need to improve cranioplasty techniques during World War II, and it is believed that Zander et al. were the first to use PMMA in patients [5]. Since then, the use of PMMA has been extensively studied and reports of long-term experience with this material are accumulating, making it well established in clinical practice [6]. Despite conflicting data in the literature regarding infection rates, it has remained a commonly used material to date. For example, a systematic review comparing titanium mesh, PMMA, polyetheretherketone (PEEK), and Norian implants states that PMMA implants are associated with a significantly higher infection rate [7]. Indeed, PMMA and autologous bone have been reported to have significantly higher infection rates than titanium mesh [8]. However, comparing preformed PMMA implants with intraoperatively freehand-molded ones, the preformed implants have lower infection rates and are comparable to autologous bone grafts [9]. Preformed titanium implants also have lower infection rates than intraoperatively freehand-molded PMMA implants [10]. This underlines one of the advantages of extracorporeal implant molding and leads to the production of safer implants. However, a meta-analysis showed no significant differences between PMMA, titanium mesh, and autologous bone in terms of overall complications such as infection, hematoma, dehiscence, seroma, material displacement, thermal sensitivity, bone resorption, and edema [11]. To overcome some of these complications, studies have also been conducted on coating PMMA implants to promote osseointegration of the outer surface regarding long-term fixation, implant loss rate, and morbidity [12]. Additionally, various modifications have been made to PMMA cements to provide an antibacterial effect, often associated with an initial release after implantation. Therefore, studies with new bioactive particles have been proposed to prevent infections with long-term effects [13,14]. Chronic irritation or delayed hypersensitivity reactions with PMMA have also been reported, likely due to non-polymerized residual components that are highly allergenic [15]. Residual monomers can cause toxic remote reactions leading to neurotoxicity [16]. A PMMA implant polymerized outside the body has fewer monomers and thus a lower allergenic potential. Additionally, brain and bone thermal damage could be due to heating during polymerization [17]. This extracorporeal workflow could avoid these complications.

Due to the advancements in three-dimensional (3D) printing, new methods are emerging that can further improve other techniques established in clinical practice. Conventional freehand-molding lacks precision, limiting its esthetics, and, in addition, a constant implant thickness cannot be ensured, thus altering the biomechanics. Therefore, to achieve the best possible esthetic and functional outcome, the use of virtual planning instead of freehand-molding is recommended for cranioplasties, particularly when involving the frontotemporal region [18]. Fused filament fabrication (FFF) technology using PEEK has recently entered the market but is not widely used due to cost and handling issues. PEEK is significantly more expensive than other biocompatible materials, which increases healthcare costs and is thus not economical for daily patient-specific treatment. As a result, it has not yet established itself for routine use. The prices for PEEK implants are significantly higher than for PMMA implants and are in the upper four-digit to lower five-digit range [19]. In contrast, the prices for PMMA implants according to the workflow outlined below are on average in the lower three-digit range [20]. PEEK is highly susceptible to changes in processing conditions because of its high melting temperature and semi-crystalline nature, especially in FFF technology due to the occurrence of a thermal gradient [21]. Due to processing, discrepancies between bulk crystallinity and linear crystallinity may be reflected in properties, so more knowledge is needed to improve the manufacturing process for safe implants [22]. Currently, PEEK is mainly in the development stage for in-house implant manufacturing [23,24]. The process of ordering a patient-specific implant (PSI) is time-consuming, thus preventing immediate surgical treatment. A publication in 2017 described a workflow that combines the advantages of 3D printing and molding, thus incorporating the benefits without the drawbacks of either technique [20]. At present, most surgeons have basic knowledge of 3D printing and can perform elementary digital workflows. The impression technique is simple, less expensive, and allows in-house production. This workflow has proven successful in many institutions, and such reconstructions have since been performed frequently [25]. Thus, this workflow is now part of routine clinical practice. Further studies confirmed that a similar workflow could produce highly accurate PSIs with high patient satisfaction [26]. Cranial implants, which were manufactured according to the workflow proposed in this paper, had a root mean square (RMS) of 1.128 to 0.469 mm, with a median RMS (Q1 to Q3) of 0.574 (0.528 to 0.701) mm [27]. The study demonstrated that highly accurate, PSIs are achievable with this workflow.

The strength of human cortical bone varies with age, mainly due to increased porosity and changes in bone mineralization. Cranial bone consists of a complex three-layered structure. Thus, there is a correlation between cortical thinning and age for frontal, occipital, and parietal bone, decreasing between 36% and 60% with age in females [28]. The overall average thickness of the parietal bone is about 6.69 ± 0.22 mm [29]. However, race-dependent changes in parietal skull bone thickness have also been described [30,31]. The parietal bone is not considered as strong as other cranial bones and should be well protected [32]. The biomechanics of the cranial bone varies greatly depending on, for example, method of force application, impact speed, the integrity of the skull, age, sex, and race. This illustrates that biomechanical load testing is an important method that provides clues for further improvement of this workflow to ensure patient safety by preventing brain injury. For example, the implant thickness, fracture pattern, implant size, and reconstruction region are essential determinants. No standards for testing PMMA cranial implants have been established, and confirmed data on fracture toughness, and standard biomechanical tests are scarce [33,34,35,36,37,38]. Animal studies have demonstrated that PMMA provides the greatest neuroprotection in cranioplasties compared to high-density porous polyethylene and autologous bone [37]. This is consistent with the conclusion from the literature that, according to biomechanical studies, PMMA cranioplasties are suitable for reconstructing lost skull bone to protect the brain [36]. Therefore, the purpose of this study was to evaluate the resistance of template-molded PMMA PSIs to withstand centrally and vertically applied uniaxial compressive forces applied to their external surface. The biomechanical tests were carried out with 3 mm thick implants. Our clinical experience has shown that this thickness allows good handling during fabrication, sufficient fixation of the screws and no implant fractures occurred under clinical conditions. This is also consistent with the findings in the literature that a thickness of 3 mm has high compressive and flexural strength when the composition of powder and liquid is equal, similar to the present study [38]. The results will provide valuable information relating to properties for use in virtual design.

## 2. Materials and Methods

In the present study, virtually planned and template-molded PMMA PSIs were evaluated. As the core material, PALACOS R (Heraeus Medical GmbH, Wehrheim, Germany), a high viscosity bone cement, was used in the fabrication process. This bone cement is a biocompatible, medically certified material and is regularly used in reconstruction in various disciplines. Ten exemplary PSIs were fabricated under similar conditions according to a workflow published in 2017 [20]. The weight and porosity properties of the PSIs were recorded. Then, a force was applied to the PSIs using a ProLine Z050TN (ZwickRoell GmbH & Co. KG, Ulm, Germany) uniaxial professional compressive testing machine until breakage. Measurements of force and breaking patterns were recorded using the testXpert II (ZwickRoell GmbH & Co. KG, Ulm, Germany) testing software.

### 2.1. Study Protocol

**Figure 1:**
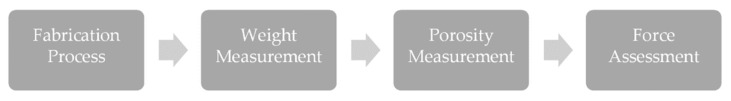
Study modules.

The protocol for this study incorporates the fabrication process, weight measurement, porosity measurement, and force assessment of the PSIs (Figure 1).

### 2.2. Fabrication Process

#### 2.2.1. Digital Data Acquisition and Virtual Planning

**Figure 2:**
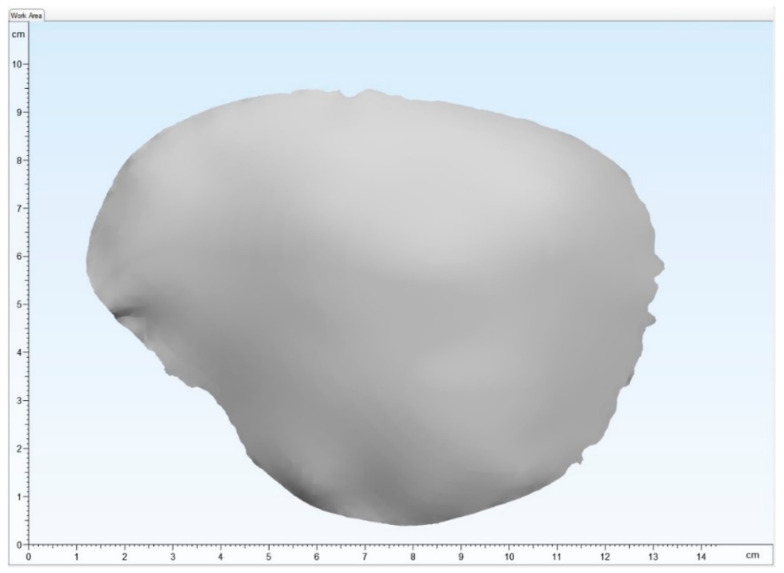
The visual output of the Standard Tessellation Language file of a patient-specific cranial plate template.

Image data from a spiral computed tomography (CT) scan of a skull with a temporoparietal defect were selected. Here, a slice thickness of 1 mm shows sufficient data quality for virtual planning. The Digital Imaging and Communications in Medicine (DICOM) data were imported into the Mimics Innovation Suite v. 20.0 (Materialise NV, Leuven, Belgium) medical 3D image-based engineering software, segmented and edited. The intact contralateral side of the bone was mirrored onto the defect area. Using a surface-reconstruction algorithm and minor adjustments, the final virtually planned template of the PSIs was acquired and exported as a Standard Tessellation Language (STL) file for the printing process (Figure 2).

#### 2.2.2. 3D Printing Process of the Template

**Figure 3:**
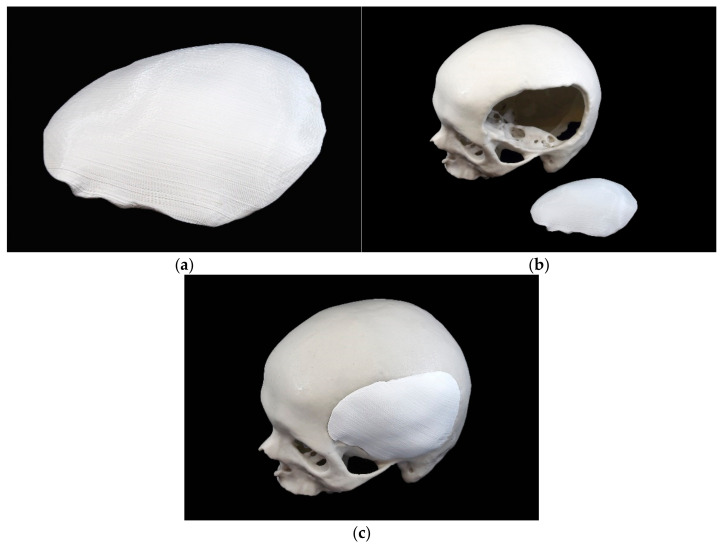
3D printed cranial plate template fabricated from polylactic acid: (**a**) trimmed template; (**b**) trimmed template in front of skull model; (**c**) trimmed template in the skull model.

The template as outlined in the STL file was printed using a MakerBot Replicator+ (MakerBot Industries, Brooklyn, NY, USA) desktop 3D printer using polylactic acid (PLA) filament (MakerBot filament true white, MakerBot Industries, Brooklyn, NY, USA) with an infill of 10% and a layer thickness of 0.2 mm. An FFF 3D printer offers high accuracy and can process low-cost printing materials such as PLA, which is suitable and sufficient for template production [39]. Subsequently, the support structures were removed, and minor imperfections were corrected in a post-processing step (Figure 3).

#### 2.2.3. Fabrication Process of the Silicone Mold

**Figure 4:**
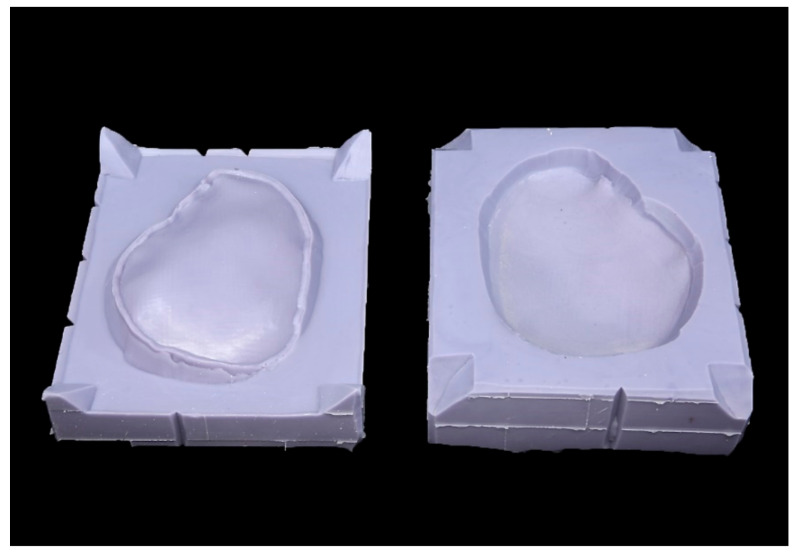
Silicone mold of the patient-specific cranial plate template.

The template was embedded in an additive silicone (Dublisil 30, Dreve Dentamid GmbH, Unna, Germany) to create two forms, a positive and a negative mold. The template had been coated with Vaseline to ensure its easy removal. Retention grooves at the edges ensured correct positioning (Figure 4).

#### 2.2.4. Manufacturing Process of the PSIs

**Figure 5:**
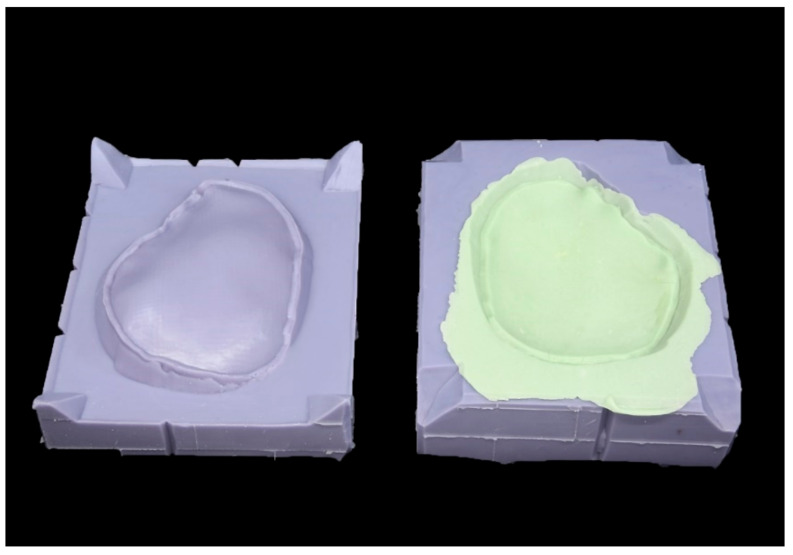
Molded patient-specific polymethylmethacrylate cranial implant with excess material.

According to the manufacturer’s instructions the molds were filled with PALACOS R bone cement (Heraeus Medical GmbH, Wehrheim, Germany). Constant pressure was applied and, after an exothermic reaction occurred, the molds were separated, and the fabricated (molded) implant was removed (Figure 5).

**Figure 6:**
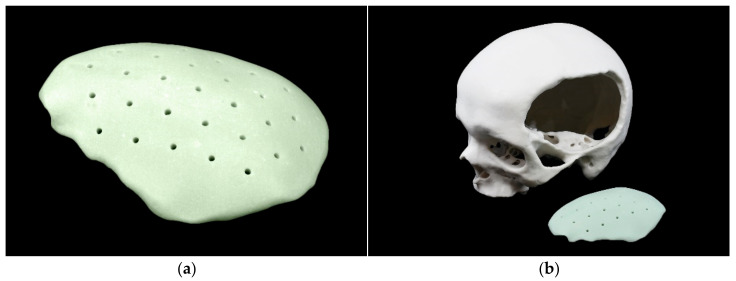

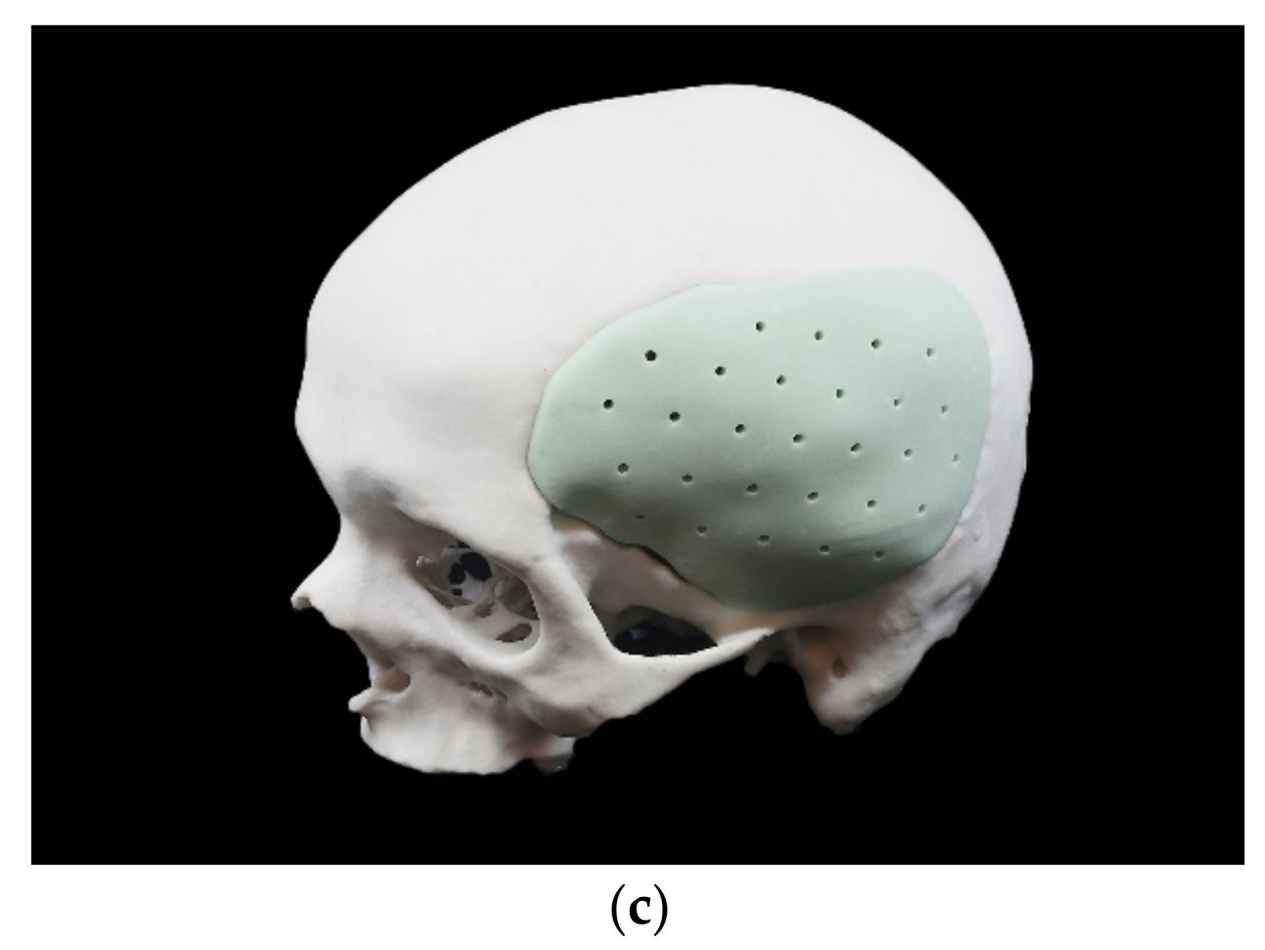
Patient-specific cranial implant fabricated from polymethylmethacrylate: (**a**) trimmed implant with drill holes; (**b**) trimmed implant in front of skull model; (**c**) trimmed implant in the skull model.

The PMMA implant was trimmed of surplus, the sharp edges removed with a pierce, and the edges smoothed using a file. Finally, holes for drainage were made using a drill (Figure 6).

### 2.3. Weight Measurement

The weight was measured with a Professional-Mini Foraco (Dongguan Shi Bo Heng Electronics Co. Ltd., Dongguan, China) precision digital scale. The measurements were performed in triplicate, and the average weight of each implant was calculated (Table 1).

### 2.4. Porosity Measurement

**Figure 7:**
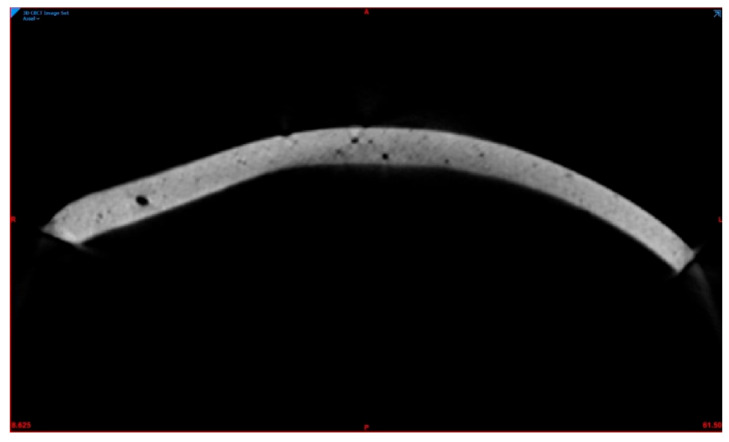
Visualization of porosities (black spots) in the patient-specific cranial implant (gray) fabricated from polymethylmethacrylate (implant no. 01).

To evaluate the porosities, the PMMA PSIs were scanned with a CS 9300 (Carestream Dental L.L.C., Atlanta, GA, USA) cone-beam computed tomography (CBCT) device using a volume of 17 × 13.5 cm^3^, 90 kV, 5.0 mA, and a voxel size of 300 µm. The DICOM data were imported into Mimics Innovation Suite v. 20.0 (Materialise NV, Leuven, Belgium), and a predefined threshold according to the density of PMMA was set to include all areas. The total volume of segmented PMMA PSIs could then be calculated. Subsequently, the individual volume of porosities for each PSI was selected, added, and subtracted from the total PSI volume (Figure 7).

### 2.5. Force Assessment

**Figure 8:**
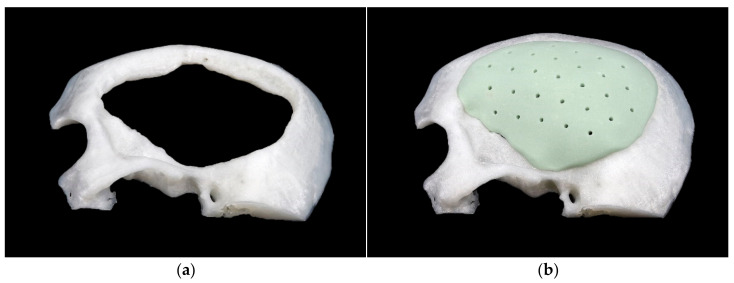
Base to prevent displacement: (**a**) without implant; (**b**) with implant.

As support construct to ensure correct, reproducible positioning without slipping off the base, the defect area was printed and mounted to the base plate of the test machine (Figure 8).

**Figure 9:**
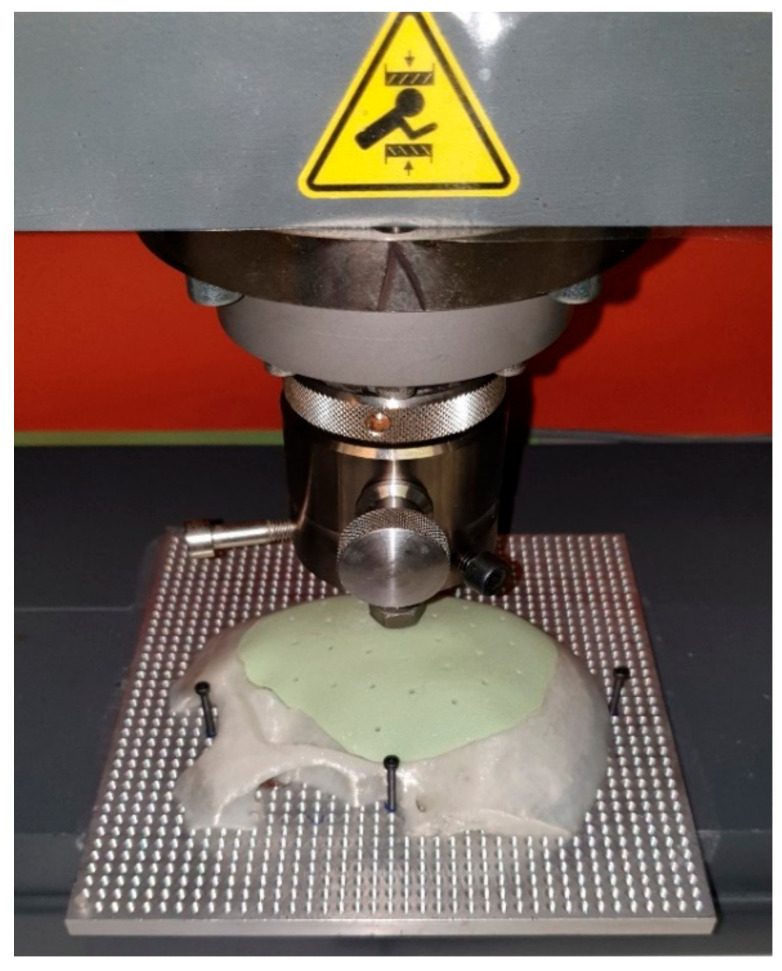
Experimental setup in the ProLine Z050TN machine.

Finally, the ten exemplary PSIs were positioned sequentially in the ProLine Z050TN (ZwickRoell GmbH & Co. KG, Ulm, Germany) material test machine. A hemispherical indenter (diameter 10 mm) was used to apply uniaxial compressive force and the implants were tested at a loading rate of 1 mm/min (Figure 9). The software recorded all relevant data, e.g., force at breakage and force at the first, second, and third crack. After the breakage of the PSIs, the different shattered parts were collected.

### 2.6. Statistical Analysis

Descriptive statistics, including mean, standard deviation (SD), median, minimum, maximum, and first and third quartiles, are reported for all outcomes. Pearson’s correlation testing was performed to assess the relationship of the applied forces with weight and porosity. Correlations were considered very weak for 0 ≤ |R| < 0.2, weak for 0.2 ≤ |R| < 0.4, moderate for 0.4 ≤ |R| < 0.6, strong for 0.6 ≤ |R| < 0.8, and very strong for 0.8 ≤ |R| ≤ 1.0. Statistical significance was set at *p* < 0.05. All statistical analysis was performed using the R statistical software (R Core Team 3.6.3, The R Foundation for Statistical Computing, Vienna, Austria).

## 3. Results

### 3.1. Quantitative Analysis

A summary of all the characteristics of this study is provided below (Table 2). The statistical results for the loading force and implant properties in terms of its weight and volume as well as the volume of porosities are reported.

#### 3.1.1. Correlation between Force and Weight

**Figure 10:**
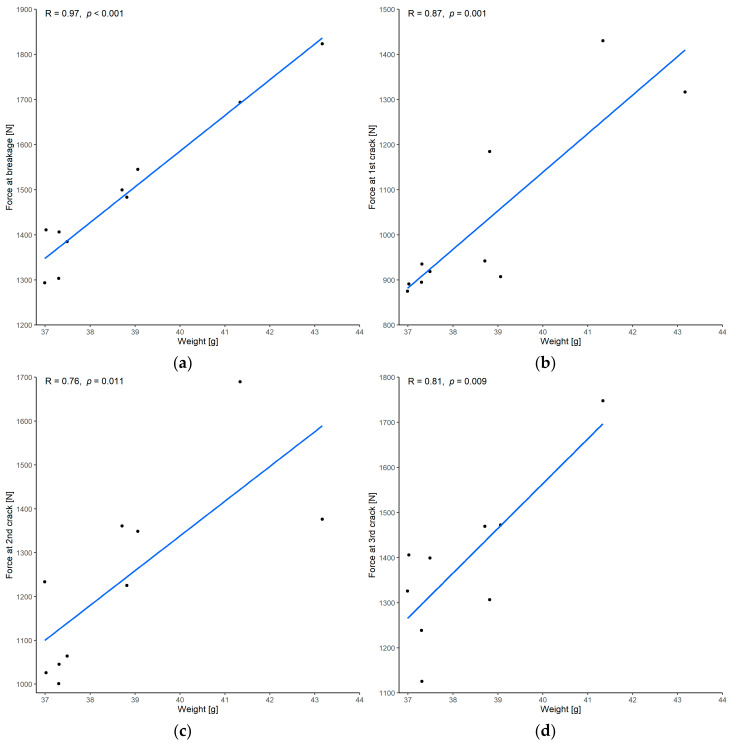
Correlation between force and weight: (**a**) force at breakage; (**b**) force at the first crack; (**c**) force at the second crack; (**d**) force at the third crack. (R = Pearson’s correlation coefficient, *p* = *p*-value of Pearson’s correlation test. One of the implants had only two cracks before it broke and was missing from the analysis of the third crack.)

There was a very strong correlation between the force at implant breakage and the implant weight, with a Pearson’s correlation coefficient of 0.97 and *p* < 0.001 (Figure 10a). Regarding the force required when the cracks occurred, there was a very strong correlation between the force at the first and third crack of the implant and a strong correlation between the force at the second crack of the implant and the weight (Figure 10b–d).

#### 3.1.2. Correlation between Force and Volume of Porosities

**Figure 11:**
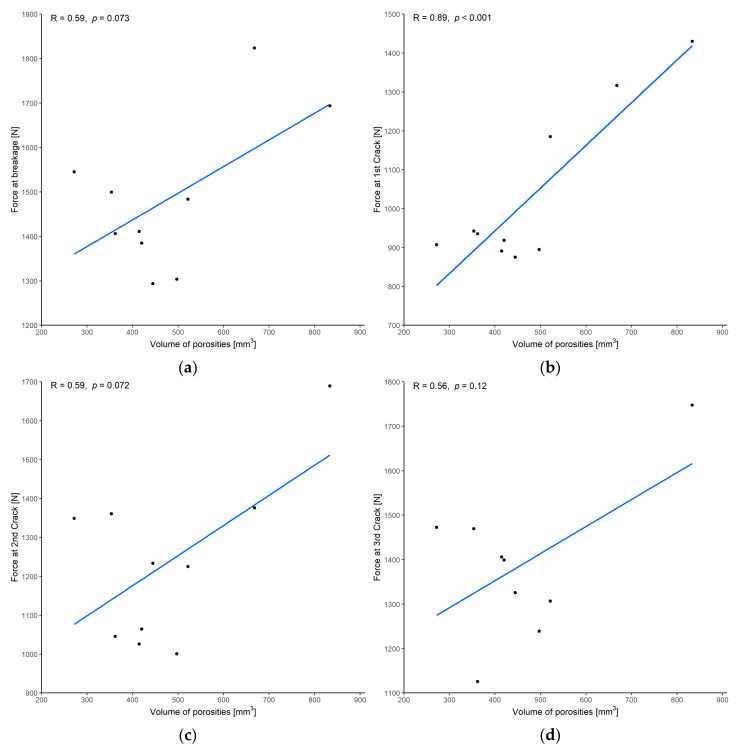
Correlation between force and volume of porosities: (**a**) force at breakage; (**b**) force at the first crack; (**c**) force at the second crack; (**d**) force at the third crack. (R = Pearson’s correlation coefficient, *p* = *p*-value of Pearson’s correlation test. One of the implants had only two cracks before it broke and was missing from the analysis of the third crack.)

There was only a moderate correlation between the force at implant breakage and the volume of porosities, with a Pearson’s correlation coefficient of 0.59 and *p* = 0.073 (Figure 11a). The correlation was not statistically significant. Regarding the force required when the cracks occurred, there was a very strong correlation between the force at the first crack of the implant, and a moderate non-significant correlation between the force at the second and third crack of the implant and the volume of porosities (Figure 11b–d).

#### 3.1.3. Correlation between Volume of Porosities and Weight

**Figure 12:**
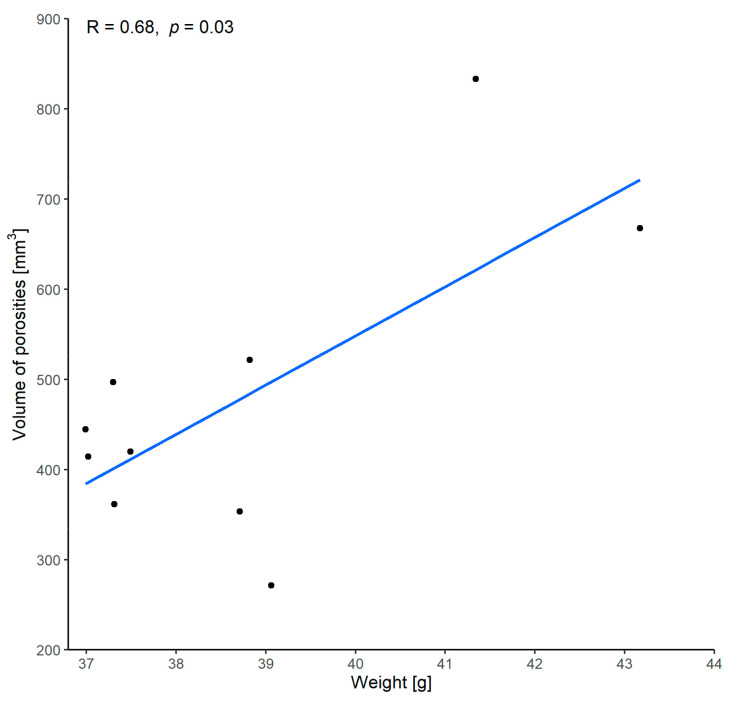
Correlation between volume of porosities and weight. (R = Pearson’s correlation coefficient, *p* = *p*-value of Pearson’s correlation test).

A strong correlation between the implant weight and the volume of the porosities was demonstrated with a Pearson’s correlation coefficient of 0.68 and *p* = 0.03 (Figure 12). This indicates that implants with higher weight also appear to have a higher volume of porosities.

### 3.2. Qualitative Analysis

**Figure 13:**
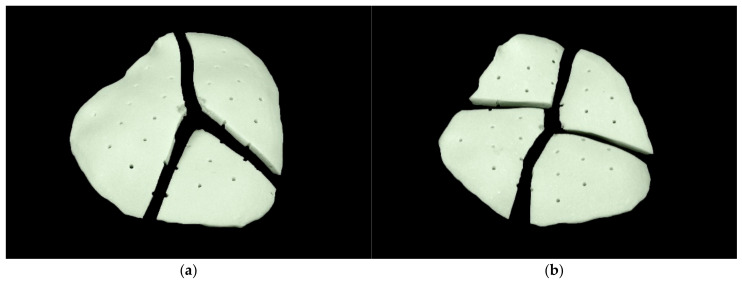

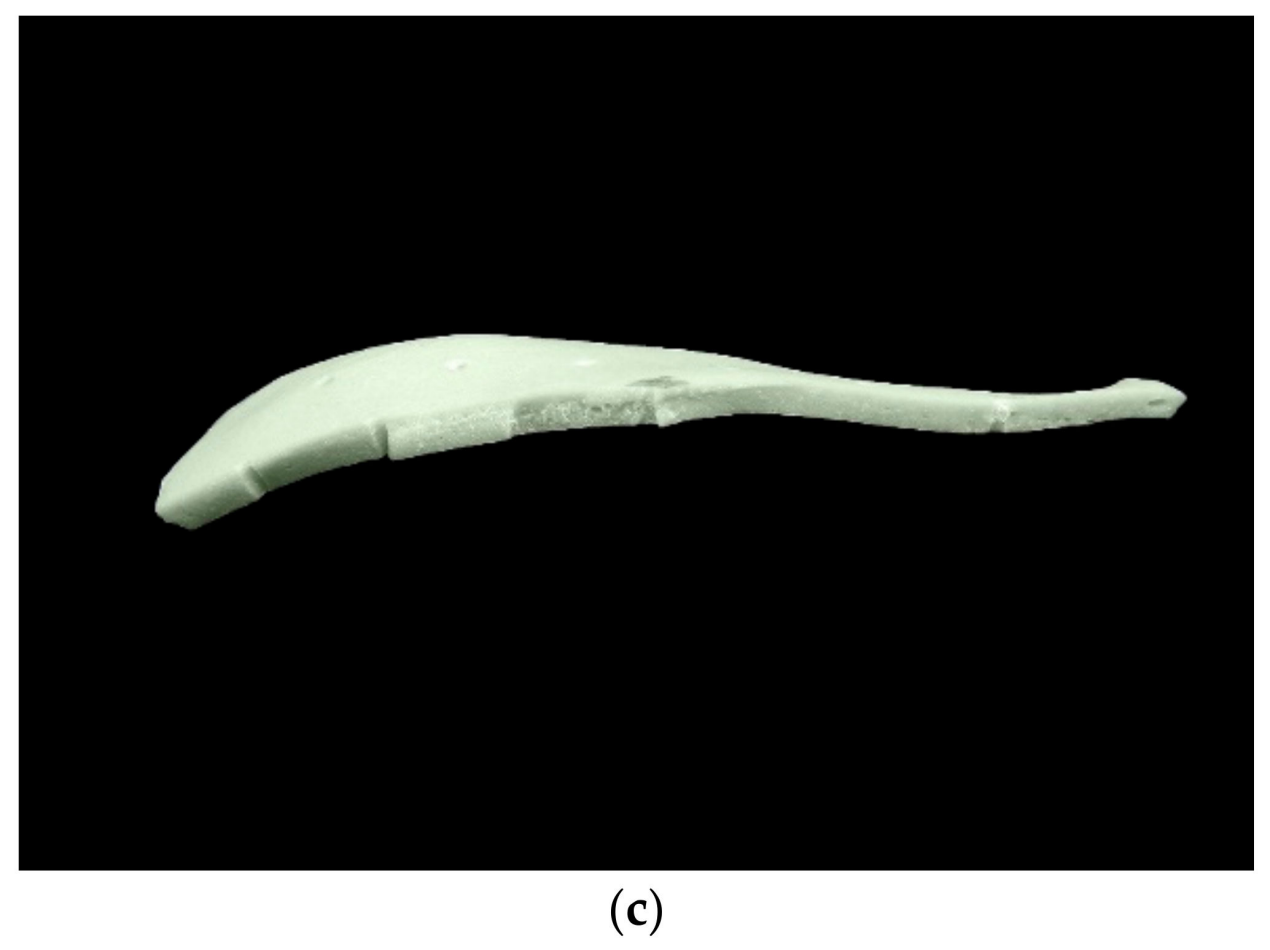
Fracture pattern of patient-specific implant fabricated from polymethylmethacrylate: (**a**) three-piece fracture pattern (implant no. 8); (**b**) four-piece fracture pattern (implant no. 10); (**c**) breaking edge (implant no. 8).

The fracture pattern showed that the cranial implants broke into no more than four parts when centrally and vertically force was applied to the external surface. Eight implants broke into three parts and two into four. The PMMA material exhibits brittle fracture behavior. A stellate fracture pattern with a rough fracture edge without splintering was observed (Figure 13). No correlation was found between the fracture pattern and the weight or volume of porosities.

## 4. Discussion

As a material for reconstructing cranial defects, PMMA has been commonly used for many decades [40]. Due to the need to achieve the most accurate anatomical reconstruction possible, the demand for PSIs is increasing. At present, patient-specific treatment is becoming increasingly central to daily medical practice. Due to virtual planning and 3D printing, new supporting techniques have emerged to ensure anatomical reconstruction and advance the available treatment options [41]. Currently, 3D printers can print implants according to the specific anatomy of the patient. However, 3D printing of PSIs from biocompatible materials is not yet widely used in clinical practice. This is mainly due to the high costs. Health insurance companies refuse to cover the costs of PSIs because current procedures do not undercut conventional implants in terms of cost-effectiveness. Therefore, a hybrid workflow was presented in 2017 that bridged the gap between 3D printing and molding and provided the cost-effectiveness and esthetics expected from conventionally prepared PSIs [20]. Moreover, an extracorporeal, template-based workflow is beneficial compared to freehand-molding and therefore remains of current interest and relevance. This hybrid workflow can significantly reduce the costs of PSIs, presenting a cost-effective alternative to conventional implants [20,42]. The material costs are very low for implant production according to this workflow. Based on current 2022 prices, the price of the PLA printing filament is less than 20 CHF/kg, the silicone for the mold is 40 CHF/L and the PALACOS R is 60 CHF/40 g. The material costs vary slightly depending on the implant size and amount to approximately 150 CHF for the procedure as mentioned above. However, manufacturing costs vary widely in terms of 3D printer, software and labor costs. Commercially available FFF 3D printers are currently available for less than 1000 CHF. The cost of the software depends on the annual license booked and can quickly reach a few thousand CHF. Economies of scale can be achieved through more frequent use, drastically reducing costs. Labor costs are also subject to strong geographic variations. However, the total cost of such an implant is a fraction of the price of a conventionally ordered, externally manufactured implant, which is in the upper four-digit range.

This study investigated centrally and vertically applied uniaxial force that a molded patient-specific PMMA implant can withstand. It is essential to determine whether a 3 mm thick implant made of PMMA can withstand sufficient force equivalent to the replaced bone to protect the brain from traumatic damage. Many studies have previously been conducted on the biomechanical properties of the skull, and accounts of a large variety of attributes can be found in the literature. The way the force is applied is one of the factors that determine the resistance of the skull. The peak fracture force for the temporoparietal bone in an intact cadaveric skull was 5633 ± 2095 N during blunt ballistic impact [43]. The mean fracture force during dynamic loading with an electrohydraulic tester was 7126 ± 1130 N [44]. In another study, the mean fracture force caused by a drop tower for the temporoparietal region with a circular plate was 5195 ± 1801 N and for the parietal region with a rectangular plate was 12,390 ± 3654 N [45]. These measurements were conducted on intact skulls with different methodological approaches however some authors have noted that the cranial strength decreases after graft harvesting from the outer or inner parietal bone layers [46,47]. As a result, the mechanical strength of the skull is reduced when bone integrity is disturbed after traumatic injury, and the fracture force is consequently lower. Therefore, PSIs should be compared with the fracture toughness of bone pieces of the corresponding region and not with values of an intact skull. The mean values and standard deviations for the maximum force of a bare skull bone piece were 728.2 ± 397.5 N with periosteum removed and 1034.0 ± 886.2 N with periosteum attached [48].

The defect investigated in the present study was a temporoparietal skull defect with a surface area of 91.87 cm^2^. This area comprises an approximately flat surface with an only slight curvature. The mean weight of the PSIs was 38.7 g, and the mean and standard deviation of force at breakage was 1484.6 ± 167.7 N. This falls within the reference range of the force for fracture of bare skull bone pieces found in the literature [48,49]. Thus, the results demonstrate that a PSI thickness of 3 mm is sufficient to resist enough force, similar to that of bare bone, to protect the brain from traumatic damage. This makes the implant significantly thinner than average parietal bone thickness, which simplifies the insertion of the implant and leaves room to compensate for cerebral edema. Planning of a thickness less than 3 mm is theoretically possible but may complicate handling, the overall impression process, and screw fixation.

The present study showed that the force resistance is proportional to the implant weight. The more material used, the higher the force resistance. Due to the manual nature of the molding process, the ten implants were not completely identical and, in some cases, had slight weight differences of a few grams. Because all of the implants had the same surface size, any increase in weight would have affected the thickness. This may be due to the varying force applied during compression of the silicone mold. This indicates that heavier implants consist of more material, resulting in greater stability, and a higher force is therefore required to break the implant. Consequently, even a small increase in implant thickness significantly increases implant resistance.

Porosities are defined as the difference between the implant volume with porosities and the implant volume without porosities. In addition to the implant thickness, the volume of porosities may also influence the mechanical stability. This study showed only a moderate correlation between force at breakage and the volume of porosities, without statistical significance. For example, implants with a higher weight also showed more porosities. The ten implants were not completely identical due to the manual nature of the molding process and, therefore, differed in porosity volume. The moderate correlation without statistical significance between force at breakage and the volume of porosities shows that implants with more porosities do not necessarily fracture more frequently. However, it is believed that higher porosity should limit fracture toughness and fatigue strength because these pores can reduce the material content along the crack plane and decrease resistance [50]. Due to porosity, the non-apparent higher susceptibility to fracture could be distorted by the minimal weight differences of the implants and the associated higher material content. Manual mixing has been described to produce types of cement with uncontrollable porosity [51]. However, the present study shows that the percentage of porosities in manual mixing is low, i.e., approximately 1–3%. Some authors recommend using a vacuum mixer to obtain the best results [52]. The manufacturer’s instructions should be followed closely to keep porosities to a minimum. Numerous factors, such as temperature, mixing time, and humidity, affect the curing time [53]. The mixing time is 30 s and should not be extended because this may increase viscosity, thus increasing the difficulty of molding. As a result, the planned thickness cannot be achieved, and the implant will become thicker as the delicate structures in the marginal areas are not reached.

Regarding the fracture pattern of the patient-specific PMMA implants, they did not shatter into many fragments upon fracture, reducing the risk of damage to underlying structures, such as brain tissue. This highlights and confirms the suitability of PMMA as a material for cranial reconstruction.

The current study is not without limitations. The duration of force application also affects stability. For example, a ballistic impact has a different effect than a slowly increasing force, as used in the present study. In this study, only forces acting vertically on the implant were measured and lateral force effects were not considered. The resistance also depends on the localization of force application. The exact region of the skull and, thus, the surface properties of the particular region will also affect stability. The surface properties of the skull differ according to increasing or decreasing curvature depending on the area. Thus, force effects on flat surfaces differ from those on curved surfaces, causing curvature effects to increase the fracture load by a factor of 2 to 3 [54]. Only the cumulative volume of porosities per implant was considered in this study, not their exact location. It is conceivable that large porosities in the area of force application promote fracture. In addition, the position of the fixation plates and screws and the placement of drill holes were not investigated. It may be beneficial to include mini-plates and screw locations in a finite element simulation to gain preoperational insight into mechanical performance, thus allowing a decision to be made about these options [33,34,35].

Due to its ease of use, practicability, and material properties, it is expected that PMMA will continue to be used in cranioplasty for many years to come. High-performance polymers such as PEEK can currently be printed, but they are uneconomical due to their high costs. As long as these new manufacturing processes remain costly, the manufacturing process described in this paper will continue to serve its purpose in daily medical applications.

## 5. Conclusions

In this study, the mean and standard deviation of the fracture force was determined to be 1484.6 ± 167.7 N for a template-molded PMMA cranial implant of the temporoparietal skull region with a thickness of 3 mm and a surface area of 91.87 cm^2^. Consequently, cranial implants that are thinner than the average parietal bone thickness while still providing equal protection for the brain can be fabricated. Thus, an implant thickness of 3 mm for a temporoparietal defect is appropriate for virtual planning, and such a constant implant thickness cannot be achieved by freehand-molding. It was determined that porosities resulting from the use of this workflow do not seem to reduce biomechanical strength. Moreover, the fracture pattern of the implants resulted in a maximum of four fragments being produced, and implants did not shatter upon fracture. These results indicate that PMMA is a suitable material for cranioplasty.

The described manufacturing process is a convenient means of fabricating template-molded PMMA implants, which are an alternative to the current, commercially available patient-specific implants. Previous studies have demonstrated this hybrid workflow’s feasibility, cost-effectiveness, and accuracy [20,27]. The outcomes of this study indicate that the PSIs produced by this process can also be assumed to be highly resistant to centrally and vertically applied uniaxial force. Thus, the current study confirmed the value of this workflow, which will continue to be favored until it is replaced by new methods, such as the likely implementation of PEEK 3D printing at the point-of-care soon.

## Figures and Tables

**Table 1:** The average weight of each polymethylmethacrylate implant.

Implant Number	Average Weight [g]
1	43.18
2	41.34
3	38.71
4	37.31
5	39.06
6	37.00
7	37.49
8	37.30
9	37.02
10	38.81

**Table 2:** Summary of all characteristics of the study.

Characteristic	Median	Minimum	Q1	Q3	Maximum	Mean	SD
Force at breakage [N]	1447.3	1293.7	1390.4	1533.8	1823.7	1484.6	167.7
Force at 1st crack [N]	927.0	875.1	898.0	1124.2	1430.2	1029.6	203.3
Force at 2nd crack [N]	1229.3	1000.9	1050.1	1357.9	1689.5	1237.0	215.8
Force at 3rd crack [N]	1399.3	1125.7	1306.9	1469.6	1747.6	1388.1	175.0
Weight [g]	38.1	37.0	37.3	39.0	43.2	38.7	2.1
Volume with porosities [mm^3^]	28,735.3	27,537.8	27,819.9	29,260.0	33,586.6	29,230.0	1946.2
Volume without porosities [mm^3^]	28,305.3	27,093.1	27,444.8	28,887.1	32,918.7	28,751.3	1831.1
Volume of porosities [mm^3^]	432.5	271.6	375.1	515.5	833.3	478.7	164.8

## Data Availability

Not applicable.

## Abbreviations

3D	three-dimensional
CBCT	cone-beam computed tomography
CHF	Swiss franc
CT	computed tomography
DICOM	Digital Imaging and Communications in Medicine
FFF	fused filament fabrication
PEEK	polyetheretherketone
PLA	polylactic acid
PMMA	polymethylmethacrylate
PSI	patient-specific implant
RMS	root mean square
SD	standard deviation
STL	Standard Tessellation Language
